# Optimized sample preparation for fecal volatile organic compound analysis by gas chromatography–mass spectrometry

**DOI:** 10.1007/s11306-020-01735-6

**Published:** 2020-10-10

**Authors:** Sofia el Manouni el Hassani, Ruud J. Soers, Daniel J. C. Berkhout, Hendrik J. Niemarkt, Hans Weda, Tamara Nijsen, Marc A. Benninga, Nanne K. H. de Boer, Tim G. J. de Meij, Hugo H. Knobel

**Affiliations:** 1 Department of Pediatric Gastroenterology, Emma Children’s Hospital, Amsterdam UMC, University of Amsterdam, Amsterdam, The Netherlands; 2 Department of Pediatric Gastroenterology, Emma Children’s Hospital, Amsterdam UMC, Vrije Universiteit, Amsterdam, The Netherlands; 3 EurofinsEAG, Eurofins Materials Science Netherlands B.V., Eindhoven, The Netherlands; 4 Neonatal Intensive Care Unit, Máxima Medical Center, Veldhoven, the Netherlands; 5 Orikami, Nijmegen, The Netherlands; 6 Philips Research, Eindhoven, The Netherlands; 7 Department of Gastroenterology and Hepatology, AG&M Research Institute, Amsterdam UMC, Vrije Universiteit Amsterdam, Amsterdam, The Netherlands

**Keywords:** Gas chromatography–mass spectrometry, Sample preparation, Volatile organic compounds, VOC, Feces

## Abstract

**Introduction:**

Headspace gas chromatography–mass spectrometry (HS-GC–MS) is widely considered the gold standard of quantitative fecal VOC analysis. However, guidelines providing general recommendations for bioanalytical method application in research and clinical setting are lacking.

**Objectives:**

To propose an evidence-based research protocol for fecal VOC analysis by HS-GC–MS, based on extensive testing of instrumental and sampling conditions on detection and quantification limits, linearity, accuracy and repeatability of VOC outcome.

**Methods:**

The influence of the following variables were assessed: addition of different salt solutions, injection temperature, injection speed, injection volume, septum use, use of calibration curves and fecal sample mass. Ultimately, the optimal sample preparation was assessed using fecal samples from healthy preterm infants. Fecal VOC analysis in this specific population has potential as diagnostic biomarkers, but available amount of feces is limited here, so optimization of VOC extraction is of importance.

**Results:**

We demonstrated that addition of lithium chloride enhanced the release of polar compounds (e.g. small alcohols) into the headspace. Second, a linear relationship between injection volume, speed and temperature, and fecal sample mass on the abundance of VOC was demonstrated. Furthermore, the use of a septum preserved 90% of the non-polar compounds. By application of optimal instrumental and sampling conditions, a maximum of 320 unique compounds consisting of 14 different chemical classes could be detected.

**Conclusions:**

These findings may contribute to standardized analysis of fecal VOC by HS-GC–MS, facilitating future application of fecal VOC in clinical practice.

**Electronic supplementary material:**

The online version of this article (10.1007/s11306-020-01735-6) contains supplementary material, which is available to authorized users.

## Introduction

Research on the potential of volatile organic compounds (VOC) as noninvasive diagnostic biomarkers has gained momentum over the past decades. VOC are carbon based volatile metabolites which can be detected in all conceivable bodily excrements (Amann et al. 2014). VOC analyses demonstrated potential as noninvasive early diagnostic biomarkers in diseases in which the intestinal microbiota is considered to play a role in the pathogenesis, such as inflammatory bowel disease (IBD), colorectal cancer, necrotizing enterocolitis (NEC) and late onset sepsis (LOS) (Berkhout et al. 2017; Berkhout et al. 2019; Bosch et al. 2019; Bosch et al. 2018; de Meij et al. 2015; van Gaal et al. 2017). In these diseases, feces is considered as the most suitable bodily excrement to be analyzed (El Manouni el Hassani et al. 2018). Fecal VOC are considered to reflect the gut microbiota composition and function, (patho)physiological metabolic processes of the host and the interaction between host and microbiota (De Lacy Costello et al. 2008).

To date, different analytical techniques have been applied to study fecal VOC, which can be divided into two main subgroups; chemical analytical and pattern based techniques. Gas Chromatography-Mass Spectrometry (GC–MS) is considered the gold standard for VOC analysis, providing an insight in the entire spectrum of VOC on molecular level (Arasaradnam et al. 2014). VOC studies are characterized by significant differences in used analytical devices, sampling methods and sample preparation, hampering the ability to compare study outcomes. Several studies, applying different analytical techniques, have demonstrated that environmental factors and sampling methods, such as sample mass, water content, duration of storage and sample temperatures significantly influence fecal VOC outcome, independent of the applied devices (Berkhout et al. 2016; Bosch et al. 2018; Reade et al. 2014).

It has been demonstrated that there are several advantages for the usage of a headspace (HS), including the simplicity of operation, degree of automation, flexibility for changing requirements, ability to control the amount of water in the HS, levels of sensitivity and quantitation (Kolb and Ettre, 2006). However, to allow for a reliable comparison between studies, and to further explore the potential of (fecal) VOC analysis in clinical practice, performing fecal VOC analysis in a standardized matter by development of a universal research protocol remains warranted.

Therefore, in the current study, optimal sampling conditions and device settings for fecal VOC analysis by means of HS-GC–MS were investigated. Study outcome may provide guidance to the development of an evidence-based research protocol on fecal VOC analysis, leading to standardization of procedures in future fecal VOC studies.

## Methods

Different groups of volatile compounds can be identified in fecal VOC mixtures. This includes acids, alcohols, esters, benzenoid, heterocyclic, aldehydes, ketones, alkanes, alkenes, alicyclic, ether, chloro, nitrogen and sulfur compounds (Garner et al. 2007). Since the abundance of alcohols were found to be different in cases compared to controls in several diseases, optimal analytical conditions were assessed in water and alcohol mixtures first (Arasaradnam et al. 2014; Probert et al. 2009). To assess optimal release of alcohols, the following variables were investigated: salt addition, injection temperature, speed and volume, septum use and the use of calibration curves. Secondly, the optimal analytical conditions were applied on fecal samples, in which the fecal sample mass was altered, and the repeatability of the method was assessed. In the next section the methods will be discussed per variable.

### GC–MS

GC–MS measurements were performed on an Agilent 7890B gas chromatograph (Santa Clara, CA, USA) equipped with an Agilent 7000C triple quadrupole mass spectrometer (QQQ MS) and a Flame Ionization Detector (FID). The QQQ MS was used in the electron ionization mode at 70 eV, with a scan range of m/z 40–300 Da and scanning rate 6 scans.s^–1^. To enable large volume injections and a pre-separation of the volatiles of interest and water a Tenax packed liner (Tenax TA (140 mg, 60–80 mesh) was used. Compounds were separated on a Restek RTX-1, 30 m × 0.32 mm × 4 μm capillary column. Software used for operating the equipment and analyzing the data was Agilent Mass Hunter (Agilent, Santa Clara, CA, USA) and the NIST mass spectral library version 2.0 build in 2012 (NIST, Gaithersburg, MD, USA). Additionally, a GC-TOF system (Agilent 7890B GC with a Time of Flight mass spectrometer and LECO Pegasus 4D system, LECO, St. Joseph, MI, USA) was used in the electron ionization mode at 70 eV, with a scan range of m/z 29–350 Da, scanning rate 15 scans.s^–1^. A Restek RTX-1, 30 m × 0.32 mm × 4 μm capillary column was used for separation of the compounds. The oven was programmed using the following temperature program: 10 °C-hold 8.22 min.- ramp 25 °C.min^−1^ to 280 °C-hold 2 min. The flow was ramped starting at 8 mL.min^−1^-hold 5.52 min.-ramp 150 mL.min^−2^ to 1.7 mL.min^−1^-hold 12.2 min. Injection was done into an Agilent multi-mode inlet (Agilent, Santa Clara, CA, USA) equipped with a packed liner filled with Tenax TA (140 mg, 60–80 mesh). The inlet was used in solvent vent mode held at 20 °C for 0.89 min.-ramped at 700 °C/min. to 250 °C-hold 6 min. Data was processed using the Chromatof software (LECO, St. Joseph, MI, USA).

### Chemicals

The chemicals that were used to evaluate sensitivity, linearity and recovery of the method can be found in Supplementary Table 1.

**Table 1:** Comparison of %RSD of a number of high abundance peaks in five fecal samples

Compound	Retention Time (s)	%RSD (area)	%RSD (area-ISTD corrected)	%RSD (area/mg)	%RSD (area/mg – ISTD corrected)
Acetaldehyde	525.4	14	9	18	12
Ethanol	605.4	16	7	19	10
2,3-Butanedione	713.7	12	4	15	7
Pentanal	791.8	13	9	15	9
Furfural	869.0	10	4	14	6
Nonanal	1004.3	22	17	24	17

Here, the relative standard deviation of the peak area of six abundant compounds are noted. Furthermore, the peak area corrected using the internal standard peak, the peak area per sample mass and the peak area per sample mass corrected using the internal standard peak are noted. The spread on the area %RSD ranges from 10—22%, when corrected by the ISTD, the %RSD values range from 4 to 9%, with the exception of nonanal (17%). By dividing the peak area by the sample mass, there is no improvement of the %RSD

*RSD* relative standard deviation, *ISTD* internal standard

### Variables assessed in mixture of alcohols and water

#### Salt addition

Salt addition has been demonstrated to enhance extraction efficiencies when added to aqueous solutions prior to solid phase microextraction (SPME) analysis (Felix et al. 1998). To enhance release of VOC into the headspace, salts and water were added to the sample (i.e. standards of alcohols in water). Hexafluoroisopropanol was used as internal standard. Four different salts were evaluated: potassium chloride (KCl), lithium chloride (LiCl), sodium chloride (NaCl) and sodium sulfate (Na_2_SO_4_). The amount of salt added to the samples was calculated using the solubility of the salts at 80 °C to create a saturated solution (Wikipedia). To 5 mL sample 2.6 g KCl, 5.6 g LiCl, 1.9 g NaCl or 2.2 g Na_2_SO_4_ was added. The influence of salt addition to the response of an alcohol mixture (C3-C10: 1-propanol, 1-butanol, 1-pentanol, 1-hexanol, 1-heptanol, 1-octanol, 1-nonanol, 1-decanol, all compounds at 400 ng.mL^−1^) in water was tested.

#### Injection temperature, speed and volume

Since the boiling point varies between volatile compounds the applied injection temperature is considered to influence the yield of measured compounds. In addition, the yield of detected compounds is known to be influenced by both the injection speed and volume. The influence of the injection temperature, speed and volume on the response (peak area) of the different alcohols was assessed by using an alcohol mixture (C3-C10) in water. The concentration of each compound was 40 ng/mL leading to an amount of 200 ng/compound per 5 mL vial. The injection temperature varied between 40 and 90 °C in steps of 10 °C. Injection speeds of 125, 250, 500 and 1000 µL/s were evaluated. The injection volume was adjusted between 0.5 and 2.5 mL in steps of 0.5 mL. The injection speed was controlled by the CTC Combipal Autosampler.

#### Septum

Three different septa were examined for their influence on the recovery of compounds, stored in a vial and present at low concentrations. The three septa assessed were a polytetrafluoroethylene (PTFE) lined septum, a barrier septum (part no. 27189, Supelco, Bellefonte, PA, USA) and a PTFE septum with an aluminum metal liner inserted at the inside. The metal liner was punched out of an Ultra High Vacuum aluminum foil and conditioned in an oven at 225 °C during 8 h while flushed with high purity nitrogen. Vials where filled with standard solutions containing acetone, toluene and chloroform in water (100 ng/mL–1 mL) and stored for 0–5 h, except for the aluminum lined septum, which was evaluated up to 8 h storage time. The recovery of the compounds was evaluated for the different storage times.

#### Calibration curves

Addition of calibration curves can aid in detailed identification of specific volatile compounds. The standards ethanol, 1-propanol, 1-hexanol, acetone, acetaldehyde, propanol, butanol, hexanol and perdeuterobenzene (C_6_D_6_) were made in demi-water. The investigated concentrations were 0, 50, 125, 250, 500, 1000 and 2000 ng mL^−1^. One mL of mixture was transferred to a glass vial in which 1.65 g LiCl was added. All samples (i.e. alcohol and water mixture) were measured in quadruplicate.

### Fecal sample mass

The fecal samples used in this study were obtained from preterm born infants selected from an ongoing multicenter prospective cohort study, in which infants born at a gestational age < 30 weeks were eligible to participate (Berkhout et al. 2017; de Meij et al. 2015). For the current study, preterm infants with NEC, LOS, spontaneous intestinal perforation or congenital intestinal anomalies (e.g. anus atresia, Hirsch sprung disease) were excluded, since it has been hypothesized that fecal VOC outcome is influenced by these entities. Feces was collected in a stool container (Stuhlgefäß 10 mL, Frickenhausen, Germany) and stored at a temperature of − 20 °C, until further handling.

The influence of fecal sample mass on VOC outcome was assessed by applying the optimal analytical conditions identified in alcohol and water mixtures. For this, one sample derived from one subject was divided into several samples with varying sample masses, which were then analyzed to assess the relation of peak area versus sample mass.

### Repeatability of the optimal analytical conditions

To assess reproducibility of the estimated optimal analytical conditions, a mixed sample was constructed by homogenizing fecal samples from six different subjects in one 10 mL glass vial. For the repeatability experiment, five samples were prepared using the final analytical conditions (200 mg fecal sample, 1.65 g LiCl, 1.25 mL water, 1.25 mL internal standard (ISTD) -200 ng/mL, injection temperature of 125 °C, injection speed 1000 µL/s and injection volume 1.5 mL) and consecutively analyzed by means of HS-GC–MS.

## Results and discussion

### Salt addition

Applying headspace technology to the GC–MS is limited in its sensitivity, due to the concentration of the VOC in the headspace. The concentration in the headspace is determined by three features; (1) the initial concentration in the sample, (2) the phase ratio between the liquid and gas phase, and (3) the partition coefficient. The latter can be influenced by the salt concentration. In a previous study by Jacq et al. it has been demonstrated that a gain in sensitivity by a factor of 2.2 was obtained by addition of salt (sodium chloride), compared to samples analyzed without salt addition (Jacq et al. 2008).

**Fig. 1:**
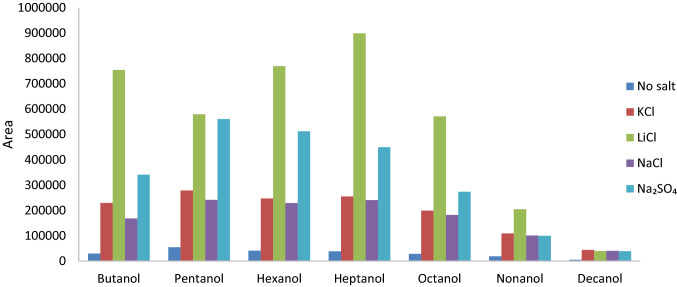
Salt addition to a water and alcohol mixture. On the y-axis the peak area is displayed and on the x-axis the different alcohols which were used as substrate of interest are displayed. Salt addition resulted in an improvement of the peak area for the alcohols assessed, in particular lithium chloride addition. An increase of the peak area up to a factor of 25 was obtained for 1-butanol, whereas for 1-decanol an improvement of 7.6 was obtained. Samples were analyzed by means of GC–MS. *KCl* potassium chloride, *LiCl* lithium chloride, *NaCl* sodium chloride, *Na*_*2*_*SO*_*4*_ sodium sulfate

In the current study, salt addition, irrespective to the type of salt added, resulted in an improvement of the peak area for the different alcohols assessed, as compared to analysis without salt addition (Fig. 1). The highest yield of VOC was obtained from samples to which lithium chloride was added, particularly for the most polar alcohols, such as butanol, hexanol, heptanol and octanol. An increase of peak area up to a factor of 25 was obtained for 1-butanol, whereas for 1-decanol an improvement of 7.6 was obtained. For low concentration samples (i.e. low concentration of alcohol in water), LiCl demonstrated to have a high background signal of VOCs derived from LiCl. Since LiCl addition resulted in an increase in yield of compounds, further experiments were performed using LiCl that was conditioned at 500 °C for a period of 14 days in a nitrogen stream, and stored in a vacuum desiccator after cooling down to room temperature (22 °C). The conditioned salt showed a strongly reduced background signal.

### Injection temperature, speed and volume

An increase in yield of the larger volatile alcohols with higher boiling points measured, was seen at higher injection temperatures. This effect was seen for 1-heptanol, 1-octanol, 1-nonanol and 1-decanol, in which the largest effect was seen for the alcohol with the highest boiling point (supplemental Fig. 1). A factor 7 in response increase was obtained for 1-decanol by increasing the injection temperature from 40 °C to 90 °C. In a previous study, in which a static headspace was used, it has been demonstrated that for medium- and low volatility solutes, the analytical sensitivity increased by increasing the incubation temperature to 70 °C (Jacq et al. 2008). Here, we used a non-static headspace in which increase of temperature to 90 °C resulted in the most optimal yield.

Increasing the injection speed from 125µL/s to 1000µL/s, resulted in an increase in the yield of measured volatile alcohols with a higher boiling point (supplemental Fig. 2). The largest effect was obtained for 1-decanol, which has the highest boiling point, where a factor 19 difference was obtained.

**Fig. 2:**
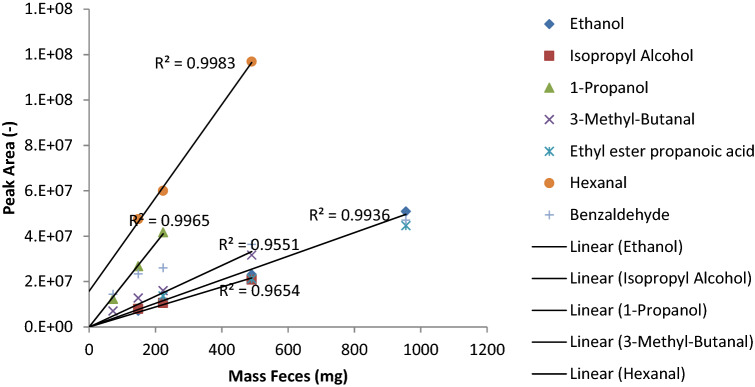
Relation between the peak area and fecal sample mass. On the Y-axis the peak area is displayed, and on the x-axis the sample mass is displayed. Here the relation between the peak area of the measured compounds (i.e. ethanol, isopropyl alcohol, 1-propanol, 3-methyl-butanol, ethyl ester propanoic acid, Hexanol and benzaldehyde) and fecal mass is plotted. For all compounds, a linear relation was observed, in which for most compounds, a fecal mass of 955 mg per sample did not meet the linear range. Samples were analyzed by means of GC–MS. *mg* milligrams; *R*^*2*^ correlation coefficient

A linear relationship between peak area and injection volume was demonstrated for injection volumes up to 2 mL (supplemental Fig. 3). After exceeding a volume of 2 mL, this effect was no longer observed, due to saturation of the detector.By increasing the injection volume, more volatiles enter the GC–MS for analyses, therefore increasing the yield of VOC measured.

**Fig. 3:**
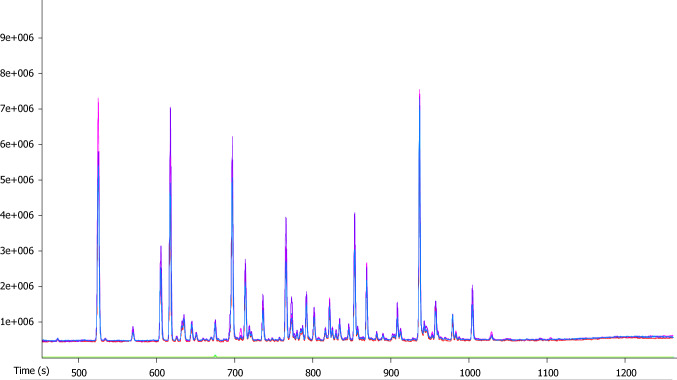
Repeatability of the optimized analytical conditions. To assess the repeatability of the optimal analytical conditions, five samples were analyzed. In this chromatogram, an overlay is displayed of the five fecal samples analyzed. It can be observed that the samples have a comparable profile. An average of 160 compounds were detected per sample. Detected compounds range from acetaldehyde (Mw = 44 g mol^−1^, T_boil_ = 20.2 °C) to 2-decenal (Mw = 154 g mol^−1^, T_boil_ = 229 °C). Samples were analyzed by means of GC-TOF–MS

Since a linear relationship between injection temperature, speed and volume up to 2 mL and the yield of alcohols is demonstrated, the highest possible injection temperature (125 °C), highest possible injection speed (1000 µL/s) and a volume of 1.5 mL were chosen as optimal analytical conditions, since this resulted in optimal signals.

### Septum

During the experiments, losses of volatile compounds were observed for low concentrations of the used standards and longer storage times of standards in vials, especially for non-polar compounds. The response of a non-polar compound, chloroform, was assessed over time using different septa (supplemental Fig. 4). We observed that using a PTFE or a barrier septum, a large loss of chloroform was perceived after storage of only a few hours. Using a PTFE septum resulted in preservation of approximately 50% of chloroform after five hours, whereas less than 20% of chloroform was perceived when a barrier septum was used. The response of the PTFE septum lined with an aluminum liner demonstrated a stable response over time, with a peak area% of 90% after eight hours storage in the vial.

### Calibration curves

Depending on the compound (all assessed compounds except acetaldehyde and hexanol), the ISTD provides an improvement on the quality of the calibration curve, yielding r^2^ > 0.996 for all compounds (supplemental Figs. 5A to 5E). For acetaldehyde and hexanol the range is limited to 1000 ng.mL^−1^ due to the non-linear response of the mass spectrometer. For the other compounds, the response was linear over the studied range (50–2000 ng.mL^−1^). Detection limits range from 3 – 24 ng/mL (LOD = 3.3 σ/S, with σ the standard deviation of lowest standard and S the slope of the calibration curve), where the LOD for acetaldehyde was 7 ng/mL, for 1-propanol 3 ng/mL, for butanol 8 ng/mL, for 1-hexanol 3 ng/mL, and for hexanol 24 ng/mL.

Use of an ISTD provided an improvement of the quality of the calibration curve, except for acetaldehyde and hexanol. For acetaldehyde and hexanol a non-linear response was seen, for the other compounds, a linear response was seen over de studied range.

### Fecal sample mass

After the optimal conditions were identified by using mixtures of water and alcohols, the optimal sampling method was assessed on fecal samples. Furthermore, the effect of sample mass on VOC outcome by applying the optimized sampling method was also assessed.

In the current study, a linear relation was observed for the abundance of the detected compounds and the fecal mass. Notably, for most compounds, 955 mg fell out of the linear range. In previous studies a similar linear relationship between compound abundance and sample mass used has been described (Berkhout et al. 2016; Bosch et al. 2018; Reade et al. 2014). This indicates that a larger sample mass results in more abundance of the compounds analyzed. Since in clinical studies performed in neonates, a limited amount of sample mass is available, we aimed at identifying an optimized sampling conditions which takes small sample size into consideration. The optimized analytical conditions, as described above, allows for sufficient VOC detection in samples with a sample mass of 200 mg.

In the following analysis, five samples, with sample mass varying between 72 to 955 mg were studied (72, 148, 223, 490 and 955 mg, respectively). For seven volatile compounds derived from a fecal sample the relation of peak area versus sample mass is plotted (Fig. 2). For all compounds, a linear relation was observed (r^2^ > 0.95) of the peak area versus the sample mass in the range plotted. For most compounds, a fecal mass of 955 mg per sample did not meet the linear range. For Hexanol, the start of the line is not from 0,0, indicating that there is possibly some Hexanol present in the background of the measurement.

### Repeatability of the method

The final sampling method has been demonstrated to be repeatable, similar amount of compounds were detected after repeating the analyses. Consequently, underlining the reliability of the here proposed sampling protocol.

Five samples taken from a homogenized sample were analyzed by applying the optimized sampling method (200 mg fecal sample, 1.65 g LiCl, 1.25 mL water, 1.25 mL internal standard (ISTD) -?200 ng/mL, injection temperature of 125 °C, injection speed 1000 µLL/s, injection volume 1.5 mL). In Fig. 3, it is displayed that the resulting chromatographs overlaid. From the overlay it can be observed that the samples exhibit a comparable profile with an average of 160 detected compounds per sample. Detected compounds range from acetaldehyde (Mw = 44 g mol^−1^, T_boil_ = 20.2 °C) to 2-decenal (Mw = 154 g mol^−1^, T_boil_ = 229 °C).

From the measured volatile compounds, six were selected which were abundant and of which the retention time of these compounds were spread over the chromatogram. The peak area of the selected compounds were used to calculate (1) the relative standard deviation (%RSD) of the peak area, (2) the peak area corrected using the internal standard peak, (3) the peak area per sample mass and (4) the peak area per sample mass corrected using the internal standard peak. The results are depicted in Table 1. The spread on the area %RSD ranges from 10 to 22%, when corrected by the ISTD, the %RSD values range from 4 to 9%, with the exception of nonanal (17%). By dividing the peak area by the sample mass, the %RSD is not improved. Table 2 depicts the groups identified in the fecal samples by using the automatic mass spectral identification of the Chromatof software.

**Table 2:** Composition of fecal sample by chemical groups

Group	Number	Area%
Acid	2	0.2
Alcohol	15	16.6
Aldehyde	22	31.3
Alkanes	7	0.4
Alkenes	6	2.0
Aromatics	7	2.5
Chlorine	3	0.1
Esters	6	2.1
Heterocyclic*	12	16.8
Ketones	8	5.2
Nitrogen	1	6.5
Sulfur	4	0.6
Silicon	2	0.2
Unknowns	56	14.8
ISTD	–	0.8

Here, the number of compounds per chemical class detected in fecal samples are displayed. In addition, corresponding peak area per chemical class are noted. Alcohols, aldehydes and heterocyclic compounds were found to be the most abundant in fecal samples

*ISTD* internal standard

^*^Heterocyclic compounds are all tentatively identified as furans

### Optimal analytical conditions

By combining the current study results, the optimal analytical conditions could be described as follows: A head space vial of 10 ml is used, a sample mass of 200 mg is recommended, in which 1.25 mL ISTD solution is added. Next, a cap using metal liner and PTFE septum is applied and put in an ultrasonic bath for 90 min. This mixture is then stored at − 18 °C. After the mixture is frozen, 1.65 g LiCl and 1.25 mL water are added and the sample can be recapped using a metal liner and PTFE septum. The sample can then be stored at − 18 °C up to analysis. For the final HS method, the following parameters are proposed to be used; Syringe = 2.5 mL-HS; Syringe Temperature = 125 °C; Flush Time = 180 s; Incubation Temperature = 125 °C; Incubation Time = 30.00 min; Agitator Speed = 250; Agitator On Time = 60 (seconds); Agitator Off Time = 10 (seconds); Injection Volume = 1.5 mL.

## Conclusion

Previous studies have demonstrated that fecal VOC outcome is influenced by a variety of variables, underlining the importance of a standardized study protocol implementable in VOC research field. Therefore, this study was performed in order to identify optimal analytical conditions to enhance the yield of analyzed VOC from fecal samples. This is particularly important when a limited sample mass is available, and therefore a limited amount of VOC can be captured. In the current study, we found that VOC outcome can be optimized by salt addition, adjustment of the injection temperature, speed and volume, usage of a PTFE septum lined with an aluminum liner. This optimized analytical conditions resulted in enhanced signal in fecal samples with a sample mass of 200 mg.

## Electronic supplementary material

Below is the link to the electronic supplementary material.

Supplementary file1 (DOCX 16 kb)

Supplementary file2 (DOCX 16 kb)

Supplementary file3 (DOCX 16 kb)

Supplementary file4 (DOCX 14 kb)

Supplementary file5 (DOCX 196 kb)
