# Supplementary material for: Optimized sample preparation for fecal volatile organic compound analysis by gas chromatography–mass spectrometry
Source: Metabolomics. 2020 Oct 10;16(10):112. doi: 10.1007/s11306-020-01735-6 (PMC7547966; doi:10.1007/s11306-020-01735-6)
Supplement: Supplementary file 1 — Supplementary file1 (DOCX 16 kb) [file 11306_2020_1735_MOESM1_ESM.docx]

Supplemental Figure 1.

**Supplemental Figure 1.** *Influence of the injection temperature on a water and alcohol mixture.* On the y-axis the peak area is displayed, and on the x-axis the assessed alcohols are displayed. An increase of the peak area is demonstrated for 1-heptanol, octanol, nonanol and 1-decanol, in which the largest effect was seen on the alcohol with the highest boiling point. The response of 1-decanol increased with a factor 7 by increasing the injection temperature from 40°C to 90°C. Samples were analyzed by means of GC-MS.
