# Supplementary material for: Optimized sample preparation for fecal volatile organic compound analysis by gas chromatography–mass spectrometry
Source: Metabolomics. 2020 Oct 10;16(10):112. doi: 10.1007/s11306-020-01735-6 (PMC7547966; doi:10.1007/s11306-020-01735-6)
Supplement: Supplementary file 2 — Supplementary file2 (DOCX 16 kb) [file 11306_2020_1735_MOESM2_ESM.docx]

Supplemental Figure 2.

**Supplemental Figure 2.** *Influence of injection speed on a water and alcohol mixture.* On the y-axis the peak area is displayed, and on the x-axis the assessed alcohols are displayed. Increase of the injection speed from 125µl/s to 1000µl/s, resulted in an increase of the peak area of butanol, pentanol, hexanal, heptanol, octanol, nonanol and decanol. The largest effect was demonstrated for 1-decanol, where a factor 19 difference was obtained*.* Samples were analyzed by means of GC-MS.
