# Supplementary material for: Optimized sample preparation for fecal volatile organic compound analysis by gas chromatography–mass spectrometry
Source: Metabolomics. 2020 Oct 10;16(10):112. doi: 10.1007/s11306-020-01735-6 (PMC7547966; doi:10.1007/s11306-020-01735-6)
Supplement: Supplementary file 3 — Supplementary file3 (DOCX 16 kb) [file 11306_2020_1735_MOESM3_ESM.docx]

Supplemental Figure 3.

**Supplemental Figure 3.** *Influence of injection volume on a water and alcohol mixture.* On the y-axis the peak area is displayed, and on the x-axis the assessed alcohols are displayed. Increasing the injection volume from 0.5 to 2.5mL results in an increased peak area, in particular for pentanol, hexanal, heptanol and decanol. For butanol, octanol and nonanol, this increase is obtained until a volume of 2mL, after this, the peak area decreases. Samples were analyzed by means of GC-MS.
