# Supplementary material for: Optimized sample preparation for fecal volatile organic compound analysis by gas chromatography–mass spectrometry
Source: Metabolomics. 2020 Oct 10;16(10):112. doi: 10.1007/s11306-020-01735-6 (PMC7547966; doi:10.1007/s11306-020-01735-6)
Supplement: Supplementary file 4 — Supplementary file4 (DOCX 14 kb) [file 11306_2020_1735_MOESM4_ESM.docx]

Supplemental Figure 4.

**Supplemental Figure 4.** *Response of chloroform over time using different septa.* On the y-axis the area percentage for chloroform is displayed, and on the x-axis the time in hours is displayed. The response of chloroform on PTFE (blue line), barrier (red line) and PTFE + Aluminum liner (green line) is displayed over the exposure time. Using only a PTFE or a barrier septum, loss of chloroform is perceived after storage of only a few hours, respectively 50% and 20% after five hours. Around 90% of the chloroform is perceived when a PTFE septum is combined with an aluminum liner for a period of eight hours. Samples were analyzed by means of GC-MS. **Abbreviations:** PTFE, polytetrafluoroethylene; Al, aluminum.
