# Supplementary material for: Optimized sample preparation for fecal volatile organic compound analysis by gas chromatography–mass spectrometry
Source: Metabolomics. 2020 Oct 10;16(10):112. doi: 10.1007/s11306-020-01735-6 (PMC7547966; doi:10.1007/s11306-020-01735-6)
Supplement: Supplementary file 5 — Supplementary file5 (DOCX 196 kb) [file 11306_2020_1735_MOESM5_ESM.docx]

Supplemental Figure 5


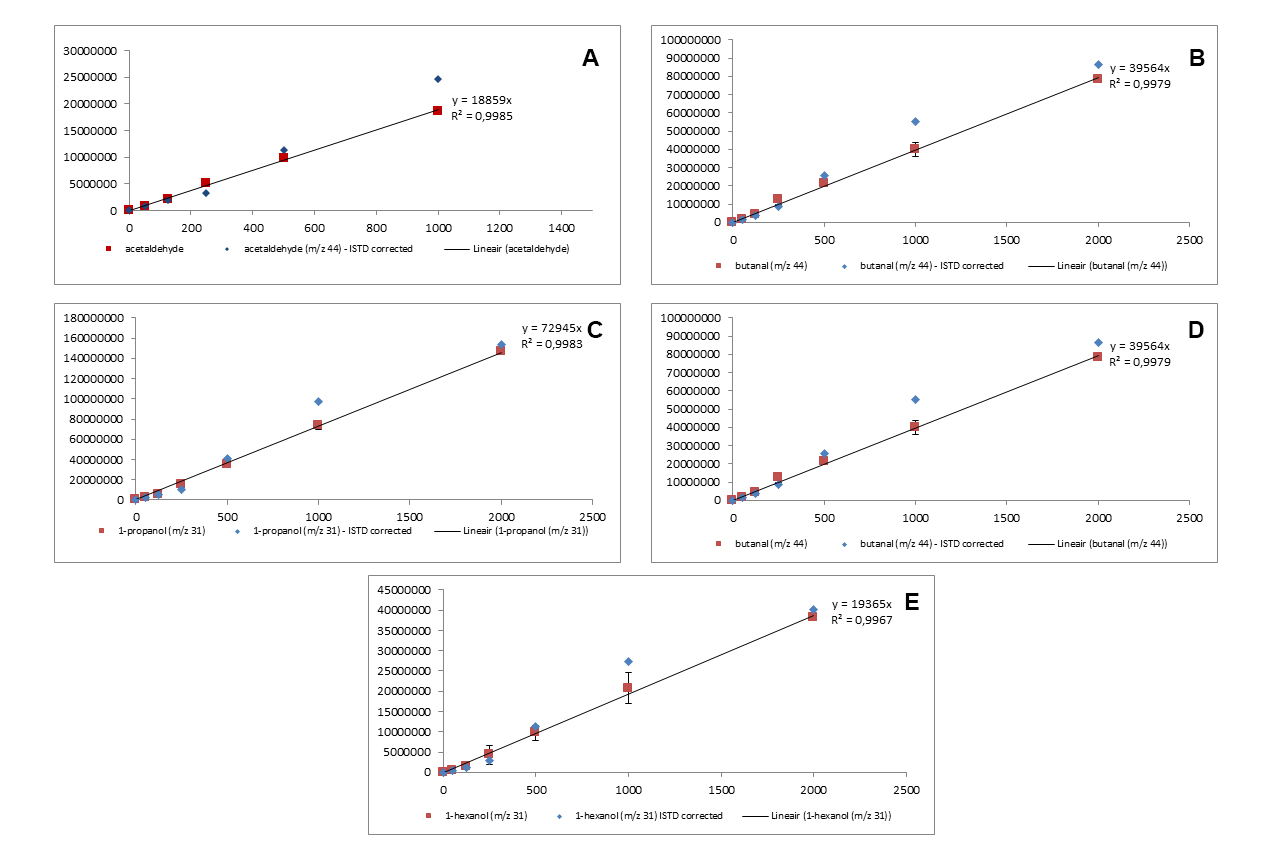


**Supplemental Figure 5.** *Calibration curves of the internal standards.* In graphs 5A to 5E a linear relation is displayed for the area measured (y-axis) and the concentration of the alcohol in water (x-axis). Acetaldehyde (m/z 44) is displayed in Figure 5A, butanol (m/z 44) is displayed in Figure 5B, 1-propanol (m/z 31) is displayed in Figure 5C, 1-hexanol (m/z 31) is displayed in Figure 5D and hexanol (m/z 44) is displayed in Figure 5E. For all compounds plotted, with the exception of acetaldehyde and hexanol, the use of an internal standard results in an improvement on the quality of the calibration curve. For acetaldehyde and hexanol the range is limited to 1000 ng.mL^-1^ due to the non-linear response of the mass spectrometer. Samples were analyzed by means of GC-TOF-MS.
